# Depth-oriented group psychotherapy for moral injury with military veterans: relational psychoanalytic and psychodynamic theory, mechanisms of action, and clinical implications

**DOI:** 10.3389/fpsyt.2026.1763407

**Published:** 2026-04-20

**Authors:** Sheila O’Brien, Ghislaine Boulanger, Jonathan Yahalom, Seamus Bhatt-Mackin, Marianne Goodman

**Affiliations:** 1 Veterans Integrated Service Network (VISN)17 Center of Excellence for Returning War Veterans, Waco, TX, United States; 2 Central Texas Veterans Healthcare System, Temple, TX, United States; 3 Private Practice, New York City, NY, United States; 4 New York University, New York City, NY, United States; 5 Health Services Research & Development, US Department of Veterans Affairs, Los Angeles, CA, United States; 6 Department of Psychiatry and Biobehavioral Sciences, David Geffen School of Medicine, University of California, Los Angeles, Los Angeles, CA, United States; 7 Veterans Affairs (VA) Mid-Atlantic Mental Illness Research, Education, and Clinical Center, Durham, NC, United States; 8 Department of Psychiatry and Behavioral Sciences, Duke University, Durham, NC, United States; 9 Bronx Mental Illness Research Education and Clinical Center (MIRECC), James J (JJ) Peters Department of Veterans Affairs Medical Center (VAMC), Bronx, NY, United States; 10 Department of Psychiatry, Icahn School of Medicine at Mount Sinai, New York City, NY, United States

**Keywords:** depth psychotherapy, group psychotherapy, military/veteran, moral injury, relational psychoanalysis

## Abstract

Some veterans are haunted by memories of action they have taken or betrayals they have experienced that violated deeply held moral beliefs; these experiences can lead to moral injury. We have developed a depth-oriented group psychotherapy for U.S. combat veterans, to address moral injury. Depth psychotherapy is an evidence-based form of psychoanalysis; the treatment we have developed is based on Relational psychoanalysis. The aim is for the group members to each develop an organized narrative about morally injurious events and their impact on their current lives to facilitate psychosocial recovery. The hypothesized change agents of this treatment are, in order of their use in the sessions: (1) warm-up team-building activities such as exercises from the improv and psychodrama/sociometry traditions; (2) reflective listening and speaking; (3) sharing moral injury event narratives with trusted others. The clinical model we have developed for treating moral injury emphasizes that veterans will be asked to describe, to the extent that they are able, the feelings, sensations, and fragmentary thoughts that are initially hard to articulate and sometimes difficult to recall. The goal of this article is to describe relevant depth psychology theory, its application to the moral injury context, the relevance of depth-oriented group psychotherapy for moral injury and, further, the depth-oriented group psychotherapy approach we have derived from these ideas.

## Defining moral injury

In recent years, researchers and clinicians treating veterans have pointed to limitations in the Post Traumatic Stress Disorder (PTSD) diagnosis (1–4). PTSD, by definition, is contingent on a traumatic stressor such as combat, where the threat of annihilation, of immediate and sudden death, of seeing others maimed or killed and by the intrusive memories to which those events gave rise (5). However, many veterans are haunted by remembering things they did, (or failed to do), “that transgress[ed] deeply held moral beliefs and expectations” or by memories of betrayals by other people or institutions for which they had previously had respect and, often, on whom their lives had depended (6). Many events that give rise to military-related moral injury are less obviously catastrophic but nonetheless violate long-held moral beliefs. These include: participating in excessive violence, engaging in revenge or retribution, seeing civilians being treated more harshly than necessary, or making decisions with inadequate information or power (7). As Jonathan Shay puts it, PTSD, as officially defined, “neatly captures the persistence into life after mortal danger and the valid adaptations to the real situation of other people trying to kill you. But PTSD is rarely what wrecks veterans’ lives, crushes them to suicide, or promotes domestic and/or criminal violence, but moral injury does” (8).

Moral injury describes functionally impairing alterations in perceptions of oneself and others, moral thinking and emotions, social behavior, and beliefs about the meaning and purpose of life, as well as increased self-harm and self-sabotaging behaviors (6). Moral injury affects the subjects’ sense of relationships: the relationship with oneself (a sense of pride or shame) with others (friendly or antagonistic feelings), and relationships with authority figures (whether they are they trusted or distrusted). In fact, the occurrence of many war-related morally injurious events involve negative changes in the subjects’ relationship to others because of having internalized group-based ‘kill or capture’ attitudes, from emotional numbing and anger as a result of grief over fallen comrades, from having dehumanized the enemy, or from failures of military leadership (7). Veterans with moral injury may describe feeling significant shame and social withdrawal, as well as anger, guilt, poor self-understanding, and existential dread (9–11).

Many morally injurious events involve real or imagined harm to others and so the relational nature of these experiences may account for the prominence of shame in the clinical picture of moral injury (12). Shame, in general, is a self-conscious affect that regulates interpersonal relationships with others and with social groups (13); it “is one’s own vicarious experience of the other’s scorn [and … ] it is an internal colloquy, in which the whole self is condemned” (13, 14). Shame can be distinguished from guilt, which, in general terms, is a negative self-conscious social emotion that focuses on one’s specific actions and their aftermath (15). Although morally injured veterans may also subjectively experience guilt alongside their shame (11), shame likely predominates because the core of moral injury is “the loss of kinship, pride in self and others, and caring and trusting relationships” (6).

Notably, emotional confusion is common and, in the aftermath of potentially morally injurious events, may be an important marker or early ‘sign’ of moral injury (10). For instance, in clinical settings, it is not uncommon to hear combat veterans say that they ‘got by’ for about 10 years post-deployment (sometimes by fighting or drinking and sometimes by over-working). However, after a seemingly dormant period, horror and shame over their wartime experiences floods in and the distance between them and other people widens.

Currently, no clinical trials of psychotherapies have specifically targeted moral injury as the focal outcome (16, 17). This is a major gap in care. And, to address it, novel psychotherapy treatments for moral injury are being developed and tested. Broadly speaking, most moral injury treatments are secular or spiritually-integrated cognitive-behavior individual or group psychotherapies (16, 18–21). Interested readers are referred to Walker and colleagues (2024) and Evans and colleagues (2023) for narrative reviews of emerging moral injury therapies. In general, these treatments aim to redress the negative impact that potentially morally injurious events have on veteran’s sense of themselves, others, and the world via cognitive restructuring activities and behavioral activation via prosocial activity planning. Imaginal exposure to morally injurious traumatic memories are components of some treatments such as individually delivered Adaptive Disclosure (22) and the group-based Moral Engagement Group (23), but not most. Evidence-based PTSD treatments, such as Cognitive Processing Therapy or Prolonged Exposure, have been recommended for moral injury, with some adaptation (24). In general, currently available treatments are limited because: 1) adapted PTSD treatments may overfocus on the present and do not address the ‘root causes’ of morally injurious shame, guilt, and inner conflict (25); 2) many treatments do not directly address the social harms entailed by morally injury either because they are delivered in an individual format or in a group format that does not support interpersonal peer-to-peer processing of morally injurious experiences and their impact. In general, treatments that aim to intervene in a veteran’s interpersonal world may best address the predominant shame and trust violation-related morally injurious harms if the treatment uses an interpersonal approach (6).

We have developed a depth-oriented group psychotherapy for U.S. combat veterans, to address the difficulties in relating to themselves and others that moral injury entails. This approach involves talking to other veterans directly about morally injurious experiences they have had. We ask each group member to develop an organized narrative about ‘what happened’ and its impact on their current lives. In general, therapeutic exposure to traumatic memories facilitates psychosocial recovery (26, 27). Our approach to this evidence-based principle of change is rooted in Relational psychodynamic theory developed to treat Adult Onset Trauma (28). The goal of this article is to describe relevant Relational and group theory, its application to the moral injury context, and describe the depth-oriented group psychotherapy approach we derived from these ideas.

## Relational psychoanalysis and the dynamics of moral injury

Beginning in the mid-1980s, the Relational School of Psychoanalysis has its roots in British Object Relations Theory and the Interpersonal School in America (29). By 2025, Relational Psychoanalysis has become an international movement with training institutes throughout the U.S, Europe, and beyond (30). Stephen Mitchell (31) proposed that the mind should no longer be viewed as a predetermined structure emerging from inside individuals, as in the classical Freudian model; rather, in his Relational model, the mind is constituted by transactional patterns acquired from individuals’ earliest relationships. Evolving relational patterns, built on and informing later patterns, form minds that are constantly in relation to others. Kulka (32) captures the fundamental paradox of selfhood when he points out that “the *raison d’etre* of the human being is not interrelating but the creation of experiences of significant selfhood—even if this goal can only be realized within the contextual cradle of relating with another.” It is no longer questioned that psychological development occurs within a relational matrix; the sense of a core-self, of self-versus-other, of self-as-initiator, and self-as-a-coherent-whole moving through time – are acquired relationally. We add to this understanding that subjective experience is a “continual process to be engaged intersubjectively” (33). In brief, the Relational model is based on the premise that human relatedness seeks to preserve the continuity, connections, and familiarity of one’s personal world.

Drawing on her research with Vietnam combat veterans and survivors of other traumatic events in adult life, Boulanger (28, 34) proposed a Relational theory of adult-onset trauma in which a catastrophic event or series of events can lead to a cascade of biobehavioral and fundamental psychological changes. The senses of agency, physical cohesion, continuity, affectivity (as they are described by Stern; 35) are undermined by terror. However, unlike posttraumatic stress disorder, which, by definition, is contingent on a traumatic stressor, where the threat of annihilation and sudden death can lead to the collapse of the self, the inception of moral injury is less obviously catastrophic. Often, without consciously articulating this belief, veterans haunted by memories of morally injurious events (as they are described in the previous section) discover over the course of time that they no longer have faith in a moral self, around which a more or less stable sense of identity had depended; further, their trust in others’ integrity is thrown into doubt. Questioning the fundamental trust in benign others and in the integrity of the self leads to self-doubt, self-blame, shame, guilt, fear of humiliation, distrust and suspicion of others, all of which contribute to a reluctance to discuss these events. These accumulating self-doubts undermine the ability to relate to others. Consequently, veterans living with moral injury become increasingly withdrawn from others, which leads to social isolation, chronic loneliness, depression, existential despair and suicidality (11, 36). These symptoms are all contributors to the “traumatic aloneness” that, as Ferenczi (37) wrote over ninety years ago, “causes the psyche to crack.”

What is useful and contributive about this model is that it posits moral injury in terms of states of mind (i.e., people’s mental states) – and particularly affective states – rather than intellectualized appraisals of right and wrong. Some morally injured veterans have said that they know ‘they did what they had to do’ or ‘there was no better option,’ but it still doesn’t feel right, and they feel as if they were in the wrong. It could be claimed that feeling in the wrong yet knowing their actions were permissible is not a matter of cognitive dissonance; in these cases, the affective experience (for example, the revulsion of having harmed another person) takes precedence over thinking that ‘those were your orders,’ ‘killing is different in war,’ etc (see (38) for discussion of the nature of moral judgments in war). Conceiving of moral injury as harming one’s sense of self captures a significant aspect of the phenomenological experience of moral injury.

## Depth-oriented psychotherapy, based on relational psychoanalysis, and the moral injury problem

Depth psychotherapy is an evidence-based, relatively short-term form of psychoanalysis (39). Depth psychotherapy is broadly characterized by a focus on affect and emotional expression; exploration of attempts to avoid topics or therapy activities; exploration of patterns of thoughts, feelings, and relating, and how they relate to past experiences; developing an awareness and exploring the meaning of psychological conflicts around strong affects such as aggression, dependency, vulnerability, and caring; identifying the unconscious and automatic and nonverbal behaviors with which people manage their conflicts; proposing hypotheses about the meaning that relates these aspects of the veteran’s experiences of themselves; a focus on interpersonal experiences including those in the therapeutic relationships; and interest in patients’ dreams, wishes, and fantasies (40).

The associated mechanisms of change in depth psychotherapy are broadly demonstrated to be meaningfully related to therapeutic outcomes (41). For instance, increases in insight or self-awareness are associated with improved interpersonal functioning and symptom severity (42). The therapist’s facilitation of the patient’s affective experience and expression are positively associated with psychotherapy outcomes (43). Increases in patients’ abilities to understand themselves and others in terms of mental states and subjective experiences (i.e., reflective functioning) are also associated with improved functioning (44). Many relational therapies are, by theory and in practice, not diagnostically bound or symptom focused and thus may be a good fit in the moral injury context.

## Depth-oriented group psychotherapy as a treatment approach for moral injury

As we will discuss, our treatment approach attempts to intervene on experiential shame via the group format, because the natural action urge from shame is to hide and not be seen (13). Group psychotherapy counters that action urge by asking people to show up and share deeply of themselves and thus grows the veterans’ tolerance of shame felt in the present moment (albeit about past events) (45, 46). Thus, although it is common for soldiers to suffer in silence and isolation with their secrets (47), depth-oriented group psychotherapies allow patients to counter a view that they must remain alone with their painful experiences (40, 45, 48). When group members listen to other members’ stories, noticing differences from and similarities to their own stories, they often feel more connected both to the other group members and to their common humanity. This is commonly called the “universality” therapeutic factor, described for decades in the group psychotherapy clinical literature (48). Group psychotherapy provides the potential for veterans to re-call, re-experience and then positively work through painful memories of morally injurious combat experiences. These communalizing experiences can break through isolation and, eventually, allow the group members to focus on life difficulties occurring in the present moment (45, 48).

## Our novel depth-oriented moral injury psychotherapy group

We designed a group psychotherapy to help morally injured veterans begin to identify and describe the experiences that have led to their difficulties in relating to others, to their distrust of others and themselves, and to their shame and social withdrawal; the treatment was specifically designed for U.S. combat veterans in the VA setting and was directly based on veteran and VA clinician recommendations and evidence-based psychotherapy practices (49). This closed group (i.e., no new members after the first few sessions) meets weekly for 90 minutes, for approximately 24 weeks total. The multiphasic treatment involves intensive team building (Phase 1 [Sessions 1-6]), listening to one another’s increasingly detailed narratives of morally injurious experiences (Phase 2 [Sessions 7-20]), and processing the meaning of these experiences as a group (Phase 2 & 3 [Sessions 21-24]). See **Table 1** for an overview of phase activities and goals. See **Table 2** for an overview of the general session format. The manual provides explicit guidance on how to facilitate affect-focused discussion among veterans, manage group dynamics (including the potential for scapegoating [i.e., when one group member is identified or ‘blamed’ for a problem that then exculpates other group members or the cofacilitators (50)], and track coping over the course of the group.

**Table 1:** Moral injury group psychotherapy phase overview.

Phase (Sessions)	Activities	Goal
1 Intensive Team Building (1–6)	• Get to know each other • Discuss past group experiences • Safety planning • Prepare to discuss moral injury events	• Form the group and build trust • Practice reflective listening and speaking • Take time before speaking on moral injury
2 Listening to and Speaking about Moral Injury Narratives (7–20)	• Revise group agreements • Decide on speaking order • Listen to and speak on moral injury • Process narratives	• Enable veterans to develop an organized narrative about their moral injury • Share the moral injury event narrative with trusted others • Facilitate discussion with about what happened and its impact
3 Processing and Wrapping Up (21–24)	• Prepare to end the group • Plan next steps • Close the group	• Reflect on and consolidate new learning and experiences • Imagine life going forward

**Table 2:** Moral injury group psychotherapy session overview.

Opening (5-10m):	• Review group agreements (Session 1, 2, 7) • Names and ice-breaker questions (Phase 1) • Grounding exercise (Phase 1, 2, and 3)	
Warm-Up (10-15m):	• Sociometry and improv exercises (Phase 1) • Light stretching (Phase 2 and 3)	
Collaborative Work (50m):	• Veteran-led and facilitator-managed • Phase 1 topics: past groups, coping, safety planning, preparing for Phase 2 • Phase 2: each member gets 2 dedicated sessions to share their moral injury event narrative • Phase 3 topics: grief, shame, courage, next steps	
Wrap-Up (10-15m):	• Group Session Rating Scale • Coping check-in using safety plans (e.g., triggers and skills) • Grounding exercise	Wrap-Up is consistent across Phases 1, 2, and 3

This treatment putatively improves psychosocial functioning by intervening on moral injury-related shame and distrust via improved symptom-specific reflective functioning and self-organization (e.g., self-image, and emotion regulation). The hypothesized change agents of this treatment are (1): reflective listening and speaking; (2) sharing one’s moral injury event narrative with trusted others; and (3) doing warm-up team-building activities such as exercises from the improv and psychodrama/sociometry traditions. See **Table 3** for the proposed change agents, mechanisms and facilitators of change for the treatment.

**Table 3:** Hypothesized change agents, mechanisms, and facilitators of change.

Change agent (Treatment component)	Mechanism of change	Rationale	Potential measurement scheme
Reflective Listening and Speaking (Treatment Session Overview: Reflective Listening and Speaking)	Improved symptom-specific reflective functioning	Ability to understand peoples’ actions in terms of mental states (e.g., emotions, thoughts) puts context and meaning into moral injury, to lessen anger	Moral Injury-Specific Reflective Functioning Scale (51)
Sharing Moral Injury Event Narratives with Other Group Members (Phase 2 Collaborative Work: Listening to and Speaking on Moral Injury Events)	Improved emotion regulation	Group members co-regulate each other’s emotions via validation and support	Difficulties in Emotion Regulation Scale (52)
	Improved self-image	Improved subjective sense of organization and integration of internal experiences (e.g., emotions, thoughts) and external events to remediate shame-based damage to self-image and relatedness	Borderline Symptom List – 23 (53)
	Improved self-compassion	Improved acceptance, nonjudgement of own and others’ suffering	State Self Compassion Scale-Short Form (54)
Warm-Up Exercises (Treatment Session Overview: Warm-Ups)	Group Cohesion	Sense of unity within the group Nonspecific factor most associated with positive treatment outcomes	Group Session Rating Scale (55)

‘Reflective listening and speaking’ are the terms we gave to the particular way of interacting that group members practice in Phase 1 to use when discussing their moral injury events in Phase 2 and processing them in Phase 2 and 3. Reflective listening and speaking involves verbalizing experiences that often go unformulated in day-to-day conversation. It involves keeping one foot in the ‘here and now’ to give voice to the immediate experiences in the immediate moment when speaking on what happened ‘then and there.’ Working in the ‘here and now’ means attending to and voicing the immediacy of emotional experience and expression in the moment of the therapy session (56), rather than voicing recollections of past emotional experiences. Reflective listening involves group members noticing and commenting publicly about what’s on their minds and what it’s like sharing their mind with the group, in the group, and in the present moment. The multiple goals of reflective listening and speaking are to: 1) support the attuned listening of other people who may ‘get’ one’s moral injuries; which in turn, 2) scaffold a sense of knowing and being known by other veterans and cofacilitators in the group; and, 3) to help knit together aspects of one’s mind that were undermined or fragmented by affect associated with the morally injurious experiences.

Reflective listening and speaking are hypothesized to improve veterans’ reflective functioning. Reflective functioning is the ability to understand people’s actions (ones’ own and others) in terms of mental states, such as thoughts and feelings (57, 58). In the moral injury context, improved reflective functioning will support veterans in bringing context and meaning into their understanding of their morally injurious experiences and thereby mitigate the cosmic loneliness and despair that comes with meaninglessness.

Sharing one’s moral injury event narratives with trusted others is an experience that the group slowly builds over the course of Phase 1 and is the core of this group’s psychotherapy process. The rationale is that memories can fester like the infection around a splinter if they’re stuck in the mind and not put into some kind of language (or symbol, like art). Memories of despair need to be narrated and shared – in a psychologically safe space – to be metabolized and their impact dissipated. During Phase 2, each member has two dedicated sessions to speak on their moral injury event. The first dedicated session occurs in the first half of Phase 2. After everyone has a chance to speak for the first time, one session is spent processing the experience of listening to and speaking on moral injury events and planning for the second time they will speak. Then, each member has a second dedicated session. Approximately 50–60 minutes in the middle of the 90-minute therapy is spent on the moral injury narratives during Phase 2. See **Table 2** for session overview. The way in which the veteran chooses to describe their morally injurious experiences is up to them: some focus on a single event, others may go chronologically from the beginning of a deployment or the beginning of their military service, and still others may focus on the impact on their current lives. The second presentation of the moral injury event narrative gives veterans a chance to recall details that they had not been conscious of earlier; there may be details that they want to get off their chest; they may want to retell the same event in a more organized and emotionally enriched way; or, they may take more time to ask other members for their thoughts about their experiences. Most people find a way to make good use of their time, and each person approaches this task in a person-specific way.

The process of narrating and sharing these memories is hoped to help veterans put back together dis-integrated or dis-associated parts of themselves – memories that don’t have any feelings or thoughts in them; to come to understand unexpectedly volatile behavior (like anger or impatience that doesn’t seem to fit the moment); or, thoughts (like intrusive thoughts and flashbacks) that don’t seem to make sense or fit the current situation. The idea is that, slowly and in the presence of dedicated and caring listeners, members will be able to put the pieces of these experiences back together and imbue them with the *here and now* experience of thinking about them (i.e., having them intentionally in mind with an awareness of one’s immediate emotional experiences) – we do this by encouraging reflection on the present-moment experience and personal relational experiences of group members, in the group.

Sharing and listening to one another’s moral injury narrative is hypothesized to improve emotional regulation and self-image. We take as a starting point that emotional regulatory processes are inherently interpersonal, because emotional experiences occur in contexts of social interactions or relationships (59). The group format provides opportunities to experience and practice multiple kinds of interpersonal emotion regulation (i.e., emotional regulatory processes that happen during social interactions), including emotional co-regulation, social sharing, perspective taking, and social modeling (60, 61). In particular, sharing one’s shameful self-experiences with like-minded, non-rejecting others and with a therapist who may assist the group fosters emotional linkages between two or more people, which facilitates emotional stability (62). An improved subjective sense of internal order and psychological integration of internal experiences (e.g., emotions, thoughts) and external events may help remediate shame-based damage to self-image and capacity to relate to others (57).

To facilitate the intensive teamwork required in reflective listening and sharing moral injury narratives, veterans perform trust- and cohesion-building exercises from improv theater, psychodrama, and sociometry in Phase 1 (63, 64). The goal of these exercises is to help people experience what it’s like to work together here and now’ toward shared goals. The exercises may feel silly or uncomfortable – and that’s the point: they are meant to get the group moving, talking, and acting together in a way that brings people closer. Importantly, they are purposefully designed not to be about moral injury events or distressing military experiences.

This change agent is hypothesized to develop psychologically safe group cohesion and thereby facilitate the other mechanisms of change. Group cohesion is the best nonspecific factor to explain positive outcomes in group psychotherapy – it is analogous to the treatment alliance in individual therapy (65). Psychological safety describes “the perception of the consequences of taking interpersonal risks in a particular context” (66). Group cohesion is the sense of ‘we-ness’ or group belonging that allows its members to belong without losing their sense of individuality or devolving into ‘groupthink’ (67). Because the group psychotherapy context involves “heightened self-consciousness and the presence of others,” it is likely that veterans will experience shame when narrating what they said or did or failed to do (46). A psychologically safe-enough psychotherapy group can promote and support the sharing. Further, we speculate that a cohesive and psychologically safe-enough group may facilitate healthy emotional regulation of shame through interpersonal interactions within the group (60). Without group cohesion, the psychotherapy group risks emotional invalidation and emotional disengagement, which could turn sharing narratives into telling ‘war stories’ and which would not address the underlying shame that may drive psychological isolation and loneliness.

The clinical model we have developed for treating moral injury emphasizes that veterans must try to describe the feelings, sensations, and fragmentary thoughts that are initially hard to articulate and sometimes difficult to recall. Many combat veterans have difficulty putting their finger on exactly what is bothering them – for some the psychic fracturing around morally injurious experiences and the subsequent dis-association makes recalling those experiences initially very difficult. In response, we emphasis the importance of the focus that group members give the thoughts, behaviors, and reactions that they had tried to avoid thinking about (because of the feelings which arise in response). The opportunity to watch and listen to other group members grapple with similar material prompts members to start talking about material that they have avoided sharing for many years and possibly dissociated. It is thus this process of speaking and listening with trusted others that, hypothetically, catalyzes the possibility for psychological change.

## Therapists’ role in this depth-oriented group psychotherapy

Although group therapy offers a promising path forward for veterans with moral injury, its inherent group processes necessarily confer complexity and thus potential challenges in delivering effective care. In each psychotherapy group, there is more for the group facilitator to consider than in the dyadic setting (68): more affect and anxieties to track, more interactions between members to notice, more potential for the problems of an individual to dominate or get missed, and more ethical situations to address. As a result, group psychotherapy requires all of the skills for individual therapy in addition to group-specific concepts, knowledge and skills (69), such as supporting group cohesion, group-as-a-whole conceptualization, the risks of scapegoating and accelerated traumatic cohesion, managing group members who try to monopolize group time, and intervening on microaggressions, among others (48, 70–73). Thus, a potential implementation challenge may be the training level required to effectively facilitate this group. Leaning about group dynamics and group facilitation in experiential groups is strongly recommended (74–76) Clinicians and chaplains interested in leading a depth-oriented psychotherapy group that leverages interpersonal processes as a change agent are directed to the American Group Psychotherapy Association for high-quality training resources, including opportunities for experiential and didactic learning (77).

In addition to the clinical skills required to successfully facilitate this group psychotherapy, therapists must also demonstrate strong ethical sensitivity and interpersonal self-awareness. Therapists will find themselves navigating questions of moral responsibility that bear directly on their veteran patients’ sense of self and wellbeing and so must be mindful to potential hazards around moral relativism or collusion with a veteran’s sense of badness (78). To complicate this clinical landscape, therapists may also find themselves more vulnerable to their own shame (79) – because of the impossibility of not making mistakes or feeling despondent or without hope because of the nature of the narratives veterans share with the group and then because these mistakes or falters are witnessed by a group of people who are counting on them. Therapists who undertake moral injury psychotherapy are strongly encouraged to find supervision or consultation within which they may discuss group dynamics and other issues as they arise [similar to the therapist consultation group recommended for Dialectical Behavior Therapy; (80]. If therapists can speak about their own sense of shame or failure, they may offer the group a powerful here-and-now role model for working through shame with others. Thus, the group therapists’ behavior may lay the foundation for the interpersonal emotion regulation that veteran patients may attempt with each other. Because of the risk – and advantages – in a group therapists’ personhood, it is especially important that group therapists have opportunities for learning in experiential groups.

## Discussion

As an overview, we proposed the following process model of this group psychotherapy: the psychotherapy group develops group cohesion (a strong evidence-based factor of change) which supports the potential for members to open up to others about their secrets (morally injurious experiences). This group cohesion scaffolds preliminary relatedness within the group – members use each other to become better attuned to themselves and help others understand themselves; increased self-understanding is a first step toward self-organization. The task of putting language into unformulated experiences and developing an organized narrative opens the possibility to share oneself with others. Sharing the narrative and listening to others share their narrative may reduce shame and improve reflective functioning (i.e., understanding their own and others’ behaviors and feelings in terms of states of mind). Reduced shame and improved reflective functioning support greater here-and-now awareness, which leads to greater agency and the possibility of connection with others – that is, improved psychosocial functioning.

Our treatment may be distinguished from other emerging individual and group psychotherapies focused on moral injury (16, 18, 19). Our group psychotherapy may be one of the only manualized treatments that asks veterans to talk directly about their haunting military experiences in a group setting. Currently, no evidence-based group treatment manuals include re-imagining scenes that caused significant distress (i.e., imaginal trauma exposures), because of concerns that it would be too destabilizing, although emerging evidence suggests these interventions are tolerable and effective (81). Our group psychotherapy is focused on within-group interpersonal experiences (i.e., prosocial learning (6) to scaffold intrapersonal change; most other moral injury-focused treatments focus on cognitive or behavioral strategies to elicit change (16). We do not directly address issues of spirituality or religiosity (19, 23, 82); our treatment, however, directly addresses issues of meaning and existential dread and was developed with the input of VA chaplains with the intention that they may facilitate these groups. Lastly, our treatment focuses on the subjective experience of shame and its repair via belonging and self-understanding; it does not explicitly target or discuss issues of guilt, responsibility, accountability, and forgiveness, as do other treatments, such as Trauma-Informed Guilt Reduction Therapy (TRiGR) (21) and the Impact of Killing intervention (83) – all of which may be relevant to the moral injury clinical picture. What we’re offering may be analogous to one step in a stage model of trauma treatment (84); work on guilt and forgiveness may be a necessary part of a later step of rehabilitation (6). As unique as our approach may be, this depth-oriented group psychotherapy accords well with the social-functional approach to moral injury recovery, which recommends interventions that target change that occurs in the veteran’s interpersonal world (versus solely intrapsychic world) and leverages helping others as a way of being helped (6).

**Figure 1:**
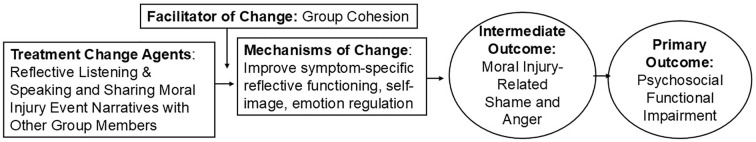
The hypothesized model of therapeutic change in the depth-oriented group psychotherapy for moral injury.

The intervention described in this article is now manualized and awaits efficacy testing. We are planning a Stage 2 (85) randomized controlled partially nested parallel-groups trial with a primary aim to test whether this depth-oriented group psychotherapy leads to greater improvement in psychosocial functional impairments related to moral injury and moral injury (9, 86, 87), compared to standard care. We will also determine whether this group leads to greater improvements in relevant intermediate outcomes – shame (88) and anger (89); and secondary outcomes – PTSD symptoms (90), pain catastrophizing (91), and psychosocial activities (92). In accordance with the National Institute of Health’s Science of Behavior Change framework (93, 94), we will also explore whether potential mechanisms of change – improved symptom-specific reflective functioning (51), self-image (53), emotional regulation (52), and self-compassion (54) – will account for change in the treatment arm but not the comparator arm. We will be directly testing whether the group psychotherapy engages the theorized mechanisms of action, and whether changes in these theorized mechanisms directly cause improvements in psychosocial functioning and moral injury. See **Figure 1** for hypothesized model of change. If the group psychotherapy is found to be not efficacious, we will be well-poised to understand why and refine and further test the intervention. If proven efficacious, this group may offer veterans an opportunity to discuss their haunting memories of war in a relationally oriented group and, ultimately, experience fuller relief from war-related psychosocial impairments. Because this group psychotherapy is founded on evidence-based processes of change, this approach may have relevance to morally injured populations beyond veterans, such as healthcare workers (95, 96) or humanitarian workers (97, 98). Future work could adapt and test this depth-oriented approach in non-combat veteran populations.

Additional next steps include conducting research on the relational dynamic model of moral injury and supporting therapists in gaining relevant clinical skills. First, our theorized model can generate a program of research to better understand the nature of moral injury within the context of relevant established psychological theories. For instance, we may be able to derive hypotheses from the social baseline theory (99) about the associations between morally injurious events that involve relational harm and/or grief and loss and subsequent psychosocial maladaptation and relationship disruption; the social baseline theory posits that the human brain assumes proximity to relationship partners and incorporates them into neural representations of the self (99). We may also be able to learn more about the nature of helpful and unhelpful therapeutic trauma exposure exercises by experimentally testing hypotheses derived from the trauma exposure continuum model (100); the trauma exposure continuum model suggests that clinical trauma exposure exercises can be dimensionally classified based on the closeness (or explicitness) of the trauma exposure and the length and duration of the exposure (the standardized cumulative dose). Future clinical research may explore the ways in which self-forgiveness obtains within our relational psychoanalytic model of moral injury (101). We also plan to develop therapist training materials that address both the relevant clinical skills as well as the ethical sensitivity necessary around issues regarding moral responsibility and the strong reactions that therapists may have during this work.

War is among the most extreme and negative human experiences and, although relational by its nature, profoundly isolates its survivors. Our novel depth-oriented approach to understanding and treating moral injury is founded on Relational Psychoanalysis and so thereby takes the relational nature of its harm and recovery as our starting place. We hope that, with current and future planned work, we may generate a better understanding and approach to moral injury rehabilitation and repair.

## Data Availability

The original contributions presented in the study are included in the article/supplementary material. Further inquiries can be directed to the corresponding author.
